# Familial variability of cerebrotendinous xanthomatosis lacking typical biochemical findings

**DOI:** 10.1002/jmd2.12197

**Published:** 2021-01-08

**Authors:** Adam J. Guenzel, Andrea DeBarber, Kimiyo Raymond, Radhika Dhamija

**Affiliations:** ^1^ Department of Laboratory Medicine and Pathology Mayo Clinic Rochester Minnesota USA; ^2^ Department of Chemical Physiology and Biochemistry Oregon Health and Science University Portland Oregon USA; ^3^ Department of Clinical Genomics and Neurology Mayo Clinic Scottsdale Arizona USA

**Keywords:** cerebrotendinous xanthomatosis (CTX), cholestanol, genotype‐phenotype correlation, spinal xanthomatosis, sterols, xanthomas

## Abstract

Cerebrotendinous xanthomatosis (CTX) is a rare autosomal recessive disorder of bile acid synthesis caused by pathogenic variants in the *CYP27A1* gene encoding the mitochondrial enzyme sterol 27‐hydroxylase. Patients with CTX can present with a wide range of symptoms, but most often have evidence of tendon xanthomas along with possible cataracts, atherosclerosis, or neurological dysfunction. Regardless of clinical phenotype, CTX patients typically exhibit levels of cholestanol and bile acid precursors in the circulation that are many fold increased over normal control concentrations. Here we report two siblings, one with the rare spinal xanthomatosis phenotype and the other with a very mild form of CTX manifesting as minor tendon xanthomatosis and gastrointestinal complaints who both carry compound heterozygous variants in *CYP27A1*: NM_000784.3: c.410G > A (p.Arg137Gln) and c.1183C > T (p.Arg395Cys). However, biochemical analysis of these patients revealed normal levels of serum cholestanol and relatively mild elevations of the bile acid precursors 7α‐hydroxy‐4‐cholesten‐3‐one and 7α,12α‐dihydroxy‐4‐cholesten‐3‐one. The atypical biochemical presentation of these cases represents a diagnostic challenge for a disorder once thought to have a sensitive biomarker in cholestanol and highlight the need for thorough investigation of patients with symptomatology consistent with CTX that includes bile acid precursor biochemical testing and molecular analysis.

**Synopsis**

Some patients with cerebrotendinous xanthomatosis (CTX) do not exhibit typical biochemical findings of elevated cholestanol, indicating a possible biochemical subphenotype of CTX with normal cholestanol levels.

## 1 INTRODUCTION

Cerebrotendinous xanthomatosis (CTX, OMIM 213700) is a rare autosomal‐recessive disorder of bile acid synthesis caused by the presence of pathogenic variants in the *CYP27A1* gene which encodes the enzyme sterol 27‐hydroxylase, a mitochondrial‐localized enzyme responsible for side‐chain oxidation required to generate bile acids. When activity of sterol 27‐hydroxylase is deficient, it's major substrate, 5β‐cholestane‐3α,7α,12α‐triol, accumulates in the liver and is metabolized by alternative pathways that result in elevated levels of C_27_ bile alcohols, detectable predominantly as bile tetrol glucuronides in plasma and bile pentol glucuronides in urine. In addition, the lack of bile acids that normally regulate the initial CYP7A1 enzyme‐mediated rate limiting step in the neutral bile acid synthesis pathway results in increased synthesis of bile acid precursors detectable in the circulation, such as 7α‐hydroxy‐4‐cholesten‐3‐one and 7α,12α‐dihydroxy‐4‐cholesten‐3‐one. The biomarker 7α‐hydroxy‐4‐cholesten‐3‐one has been used as a measure of the rate of bile acid synthesis in a number of studies.

CTX is a multisystem disorder with clinical symptoms that manifest as a result of deposition of cholesterol and cholestanol in many different tissue types, including in the lens of the eye causing cataracts, vasculature causing atherosclerosis, tendons causing xanthomas, and the nervous system causing neurological dysfunction. The age of symptom onset is variable, but can begin with cholestatic jaundice and diarrhea in infants, cataracts, developmental delay, or intractable diarrhea beginning in childhood, motor dysfunction or tendon xanthomas generally beginning in the second to third decade of life, or possibly adult‐onset neurological dysfunction.^1^ Symptoms most commonly begin to manifest in the teenage years, but many patients remain undiagnosed for an average of 16 years from the time of initial symptom recognition and CTX patients as a group are likely underdiagnosed.^2^, ^3^ A rare subset of patients manifest clinically as a spinal xanthomatosis phenotype characterized by white matter abnormalities and myelin loss in the lateral and dorsal columns of the spinal cord.^4^, ^5^

The clinical presentation of CTX, particularly tendon xanthomas, can be mistaken for sitosterolemia or the relatively more common familial hypercholesterolemia (FH) and it is important to correctly differentiate between these disorders and initiate the appropriate treatment protocol for CTX, which generally consists of chenodiol (chenodeoxycholic acid) and statin administration. These disorders can be differentiated with biochemical testing; sitosterolemia patients typically have elevated levels of plasma sitosterol and campesterol while FH patients have significantly elevated levels of cholesterol (which can be normal in individuals with CTX). Measurement of cholestanol in plasma by gas chromatography‐mass spectrometry (GC‐MS) has traditionally served as a biomarker of CTX, but it can also be elevated in other diseases affecting the liver.^6^ Additional biomarkers have been identified by assessing plasma levels of other metabolites, such as the cholesterol precursors 7‐dehydrocholesterol and 8‐dehydrocholesterol (indicators of an increased rate of cholesterol synthesis in CTX that are also elevated in Smith‐Lemli‐Optiz syndrome),^7^ as well as more specific bile acid precursor ketosterols such as 7α‐hydroxy‐4‐cholesten‐3‐one, 7α,12α‐dihydroxy‐4‐cholesten‐3‐one, and 7α12α‐dihydroxy‐5β‐cholestan‐3‐one.^8^, ^9^ Other blood biomarkers demonstrated as useful to screen dried blood‐spots for CTX include 7α,12α‐dihydroxy‐4‐cholesten‐3‐one and 5β‐cholestane‐3α,7α,12α,25‐tetrol‐3‐*O*‐β‐d‐glucuronide.^10^, ^11^ Molecular analyses of the *CYP27A1* gene can also identify potential CTX patients, with biochemical testing serving as a functional confirmation of their potential disease.

## 2 CASE REPORT

Patient 1 was a 57 year‐old woman of French Canadian, German, and Bohemian ancestry who had neurological symptoms beginning in her 30's of progressive spasticity that led to a diagnosis of primary progressive multiple sclerosis at age 38. However, at 57 years‐of‐age, the diagnosis was questioned at our medical institution and she underwent a genetics evaluation for alternate pathologies resulting in progressive spastic paraparesis. General physical examination revealed tendon deposits. Neurological examination showed severe lower extremity weakness distally and mild‐to‐moderate weakness proximally along with profound lower extremity sensory loss below the hips, necessitating full‐time use of a wheel chair. Spinal MRI revealed longitudinal T2‐hyperintensity. However, in this case no lesions were present by brain MRI. Her clinical workup consisted of biochemical testing for CTX which revealed an elevation of the cholestanol precursor 7α,12α‐dihydrocholest‐4‐en3‐one (Table 1) and molecular analysis of the *CYP27A1* gene that revealed two pathogenic variants: c.410G > A (p.Arg137Gln) and c.1183C > T (p.Arg395Cys). Both of these variants have been reported previously (Table 2), including some cases with corresponding biochemical data.^12^, ^13^, ^14^, ^15^, ^16^, ^17^, ^18^, ^19^ This patient's imaging has been described previously^20^ and is consistent with rare previous reports of spinal xanthomatosis occurring both with^4^ and without brain lesions.^21^ Upon diagnosis with CTX, a regimen of chenodiol (250 mg three times per day) and atorvastatin were initiated for the patient; however, she developed transaminitis requiring her dose to be reduced to 250 mg daily. Biochemical testing was repeated after 8 months; this time including a full sterol panel (Table 2).

**TABLE 1:** Biochemical results for two reported CTX patients

Compound	Reference range (μg/mL)	Patient 1		Patient 2
		Pretreatment	Treated (+8 mo)	
8‐Dehydrocholesterol	<2.0		0.50	**8.33**
8(9)‐Cholestenol	<5.0		0.44	0.85
Cholestanol	<5.0		2.91	2.58
Desmosterol	<2.0		0.58	0.29
7‐Dehydrocholesterol	<1.0		0.35	**10.60**
Lathosterol	<3.0		1.03	**4.54**
Campesterol	<7.0		3.89	2.85
Stigmasterol	<1.0		0.07	0.15
Sitosterol	<5.0		4.49	2.48
7α,12α‐Dihydrocholest‐4‐en3‐one	<0.100	**0.722**	**1.00**	**1.52**
7α‐Hydroxy‐4‐cholesten‐3‐one	<0.300	0.183	**0.32**	**0.60**

*Note*: Bold text indicates a value greater than the reference range.

Abbreviation: CTX, cerebrotendinous xanthomatosis.

**TABLE 2:** Characteristics of variants reported in CTX patients

	*CYP27A1* molecular variants		Cholestanol^a^	
Source	Variant 1	Variant 2	(μmol/L)	(μg/mL)
Nozue et al^13^	c.410G ≥ A (p.Arg137Gln)	c.1421G > A (p.R474Q)	19.8	7.7
Tada et al^12^	c.410G ≥ A (p.Arg137Gln)	c.410G ≥ A (p.Arg137Gln)	13.4	5.2 (<3.08)
Chen et al^14^	c.410G ≥ A (p.Arg137Gln)	c.410G ≥ A (p.Arg137Gln)	NR	NR
	c.410G ≥ A (p.Arg137Gln)	c.379C > T (p.Arg127Trp)	NR	NR
Smalley et al^15^	c.1183C ≥ T (p.Arg395Cys)	c.256‐1G > T	74.6 (>12.6)	49.4
Huidekoper et al^16^	c.1183C ≥ T (p.Arg395Cys)	c.1016C > T (p.Thr339Met)	21.5 (>19.0)	8.4
Stelten et al^17^	c.1183C ≥ T (p.Arg395Cys)	c.646G > A (p.Ala261Pro)	28.6 (>9.6)	11.1
	c.1183C ≥ T (p.Arg395Cys)	c.646G > A (p.Ala261Pro)	13.7 (>9.6)	5.3
	c.1183C ≥ T (p.Arg395Cys)	c.1016C > T (p.Thr339Met)	83 (>12.5)	32.3
	c.1183C ≥ T (p.Arg395Cys)	c.1016C > T (p.Thr339Met)	79 (>12.5)	30.7
	c.1183C ≥ T (p.Arg395Cys)	c.850A > T (p.Lys284*)	69.4 (>12.5)	27.0
	c.1183C ≥ T (p.Arg395Cys)	c.850A > T (p.Lys284*)	53.4 (>12.5)	20.8
	c.1183C ≥ T (p.Arg395Cys)	c.1184 + 1G > A	140.9 (>10)	54.8
	c.1183C ≥ T (p.Arg395Cys)	c.1184 + 1G > A	39.1 (>10)	15.2
Catarino et al^18^	c.1183C ≥ T (p.Arg395Cys)	c.1184 + 1G > A	NR	NR
	c.1183C ≥ T (p.Arg395Cys)	c.1184 + 1G > A	NR	NR
Chen et al^19^	c.1183C ≥ T (p.Arg395Cys)	c.435‐12G > T	68.4	26.6

Abbreviations: CTX, cerebrotendinous xanthomatosis; NR, not reported.

^a^ Number in parenthesis indicates upper limit of reported control range where provided in original publication.

Patient 2 was a female evaluated at 60 years‐of‐age due to her sibling's (patient 1) recent diagnosis of CTX. Her past medical history included breast cancer and a cholecystectomy due to the presence of stones in her gallbladder. She also reported bilateral swelling and stiffness of her Achilles tendons. Neither patient had cataracts as reported in a majority of CTX patients, but both did report increased GI sensitivity and intermittent diarrhea that has been, retrospectively, linked to this diagnosis. Patient 2 was found by targeted familial molecular testing of the *CYP27A1* gene to have the same two pathogenic variants identified in patient 1. A full sterol and bile acid precursor biochemical profile was utilized for patient 2 and an elevation of 7α,12α‐dihydrocholest‐4‐en‐3‐one and 7α‐hydrocholest‐4‐en‐3‐one (thought to be a cholestanol precursor) was observed, along with other intermediates of cholesterol biosynthesis (Table 1). Upon diagnosis, she was placed on chenodiol therapy at a dose of 250 mg twice daily.

The plasma cholestanol and ketosterol results for both siblings were confirmed by a second accredited laboratory offering clinical biochemical diagnostic testing. The plasma bile alcohol 5β‐cholestane‐3α,7α,12α,25‐tetrol‐3‐*O*‐β‐d‐glucuronide was also measured on a research basis using isotope dilution HPLC‐tandem MS in the sample from patient 1 on treatment (273 ng/mL) and in untreated patient 2 sample (1740 ng/mL); the concentration in unaffected individuals is normally <200 ng/mL.^10^

## 3 DISCUSSION

CTX is a rare autosomal recessive disorder of bile acid synthesis leading to abnormal lipid metabolism and storage. It is caused by pathogenic variants in the *CYP27A1* gene and subsequently decreased sterol 27‐hydroxylase activity. CTX patients are clinically heterogeneous both in terms of initial symptoms and age of presentation, making diagnosis difficult and leading to significant diagnostic delay. Due to this heterogeneity and relatively low incidence, CTX is likely significantly underdiagnosed. While the most common symptoms are juvenile bilateral cataracts, tendon xanthomas, intractable diarrhea, and neurological dysfunction, there is also a rare spinal xanthomatosis disease variant characterized by white matter changes and demyelination in the spinal column.

The two patients presented here are from the same family and noteworthy due to the very different clinical courses related to their underlying CTX disease and their very subtle biochemical findings. Patient 1 first had symptoms of spastic paraparesis beginning in the third decade of life and was diagnosed with progressive multiple sclerosis at age 38, but was later diagnosed with CTX. Similar scenarios have been documented previously where CTX patients with the rare spinal xanthomatosis phenotype carry a multiple sclerosis diagnosis for many years.^4^, ^5^ Conversely, patient 2 had a comparatively mild phenotype which consisted of moderate Achilles xanthomatosis and possible enterohepatic involvement as evidenced by gallstones requiring a cholecystectomy. These cases demonstrate the heterogeneity that exists amongst CTX patients, even within the same kindred and carrying identical *CYP27A1* variants. More notable are the biochemical findings in these patients, particularly their normal levels of cholestanol. These results are quite puzzling because elevation of plasma cholestanol levels has traditionally been viewed as a strong diagnostic indicator of CTX. It is generally accepted that in CTX patients cholestanol is elevated in circulation, tissues, tendon and brain xanthomas, and that accumulation of cholestanol is associated with the pathology of this disease. The mechanism of cholestanol accumulation in the CNS has been proposed to occur through transfer of elevated 7α‐hydroxy‐4‐cholesten‐3‐one from plasma across the blood‐brain barrier and subsequent conversion to cholestanol in the brain.^22^

Over 100 *CYP27A1* variants are known to associate with CTX disease,^23^ but at this time, there has been no correlation relating patient genotype to clinical phenotype.^24^, ^25^, ^26^ Both the c.410G > A (p.Arg137Gln) and c.1183C > T (p.Arg395Cys) variants identified in these patients have been reported in multiple previous CTX patient cases. The p.Arg395Cys variant (previously termed p.Arg362Cys) was demonstrated by Cali et al to dramatically reduce enzyme activity on CYP27A1 expression in vitro.^27^ Thirteen previous clinical cases were found where the c.1183C > T (p.Arg395Cys) variant was reported with corresponding patient cholestanol levels. The median cholestanol level for these cases was 68.4 μmol/L (26.6 μg/mL) and on average, a 5.2‐fold increased over the reported reference range value for each case. These results are consistent with the biochemical expectation of elevated cholestanol in CTX patients prior to therapy initiation.

The c.410G > A (p.Arg137Gln) variant is predicted to be deleterious by multiple *in silico* prediction tools (Align GVGD, SIFT, PolyPhen‐2) and has appeared in four CTX patient case reports with corresponding cholestanol values reported twice (Table 2). All four of the patients previously described with the c.410G > A (p.Arg137Gln) variant presented with characteristic tendon xanthomas and neurological sequelae were noted in two of the four (positive pyramidal tract signs in two patients including spastic paraparesis in one of them and dementia and cerebellar signs in the other patient at the time of publication). Although no definitive conclusions can be made based on these reported values, it is noteworthy that while the cholestanol levels were elevated in clinically identified CTX patients carrying the c.410G > A (p.Arg137Gln) variant, they appeared to be relatively mild elevations in comparison to those reported in the larger CTX patient population. The normal plasma cholestanol reference ranges for the two clinical laboratories contributing to this study are 2.7 ± 0.8 μg/mL and 2.3 ± 1.2 μg/mL (Mean ± SD). Cholestanol has been elevated at least above 5.0 μg/mL in all known clinical case reports of CTX. The lowest plasma cholestanol value measured in a clinically and genetically confirmed untreated CTX case by either of the contributing clinical labs was 8.4 μg/mL (21.6 μmol/L).^8^ Treatment with steroids was previously reported to lower plasma cholestanol in untreated CTX through an unknown mechanism and could also be a source of potential confounding biochemical results.^28^

An enzyme expression study of the c.410G > A (p.Arg137Gln) variant is beyond the scope of this case study, but warrants future investigation. Although this variant is predicted to be deleterious by multiple *in silico* prediction tools and pathogenicity supported by a number of CTX patient reports, compound heterozygosity seems to result in a milder biochemical phenotype (and potential attenuated clinical phenotype) that could be a result of partial CYP27A1 enzyme activity. The finding of a milder biochemical phenotype with normal plasma cholestanol, but associated clinical phenotype, could also have implications for CTX carriers. With 50% CYP27A1 enzyme activity, carriers are able to maintain normal plasma cholestanol, but when their bile acid synthesis pathway is stimulated they show an increase in urinary bile alcohols and a biochemical signature in plasma that is likely similar to that observed in the patients described here.^29^

The possibility of a milder form of CTX has been suggested previously and is reinforced by the likely underestimate of disease incidence through clinical case identification.^3^ It may be possible that the combination of p.Arg395Cys variant and p.Arg137Gln variant in these siblings allows for enough CYP27A1 activity (and generation of chenodeoxycholic acid) that the bile acid pathway is partially regulated and circulating cholestanol concentrations are normal. 7α‐hydroxy‐4‐cholesten‐3‐one concentrations were elevated in patient 2 (not as high as normally observed for untreated CTX cases), but interestingly 7α‐hydroxy‐4‐cholesten‐3‐one was initially within normal limits in patient 1. Transfer of 7α‐hydroxy‐4‐cholesten‐3‐one across the blood‐brain‐barrier into the CNS may still be occurring to some extent in the sibling cases described here with potential cholesterol and cholestanol deposition in the CNS; however, the circulating levels of 7α‐hydroxy‐4‐cholesten‐3‐one do not suggest a pathogenic effect from this analyte. These results suggest that there may be other factors contributing to the pathophysiology of disease in CTX, for example, high concentrations of plasma bile alcohols may contribute to defective functioning of the blood‐brain barrier as suggested by Salen et al,^30^ who found increased amounts of albumin and apolipoprotein B in the cerebrospinal fluid of eight untreated CTX patients. Therapy with chenodiol markedly suppressed plasma bile alcohol glucuronide concentrations,^31^ reestablished selective permeability of the blood‐brain barrier, and normalized the concentrations of sterol and apolipoprotein in the cerebrospinal fluid.

These cases highlight a possible deficiency with the diagnostic approach of using cholestanol as the primary CTX diagnostic marker, which has been accepted for many years. In order to identify all positive patients, including those with the biochemical phenotype described here, a metabolic panel including bile acid precursors such as 7α‐hydroxy‐4‐cholesten‐3‐one and 7α‐12α‐dihydrocholest‐4‐en‐3‐one, along with cholestanol, and potentially 5β‐cholestane‐3α,7α,12α,25‐tetrol‐3‐*O*‐β‐d‐glucuronide, is more comprehensive and offers the highest likelihood of identifying all CTX patients. 7‐dehydrocholesterol and 8‐dehydrocholesterol are additional markers that may be elevated in CTX, but they are less specific as they are also elevated in Smith‐Lemli‐Optiz syndrome.

It is uncertain what impact chenodiol treatment may have on any clinical disease progression in these cases and if therapeutic benefits outweigh risks. Elevated plasma bile alcohols, that may be detrimental,^31^ can be decreased on treatment. There has been no neurological deterioration in patient 1 while on treatment. Monitoring of any clinical disease progression in patient 2 (at a dose of 500 mg/day) may help to elucidate treatment efficacy and necessity for future patients with milder symptomatology. There is also the problem of monitoring therapy efficacy using cholestanol, the biomarker routinely utilized in treatment monitoring for CTX patients. In these rare cases of CTX with normal cholestanol a surrogate marker that is elevated in pretreatment samples, such as 7α,12α‐dihydrocholest‐4‐en3‐one will be monitored and correlated with clinical phenotypic response. Initial treatment of patient 1 with chenodiol (250 mg, three times daily) did not elicit a reduction of 7α,12α‐dihydrocholest‐4‐en3‐one. Plasma 5β‐cholestane‐3α,7α,12α,25‐tetrol‐3‐*O*‐β‐d‐glucuronide (demonstrated by Salen and colleagues to be markedly suppressed in 1 month at a high chenodiol dose of 1000 mg/day^31^) and urinary bile alcohols may also be useful parameters to measure for the evaluation of the effect of chenodiol therapy. Further monitoring will be necessary to determine optimal biomarkers to monitor treatment efficacy.

Although CTX is not currently included in the Recommended Uniform Screening Panel (RUSP) of disorders screened for in newborns, it has been nominated for possible addition to the RUSP. The biomarkers 7α,12α‐dihydroxy‐4‐cholesten‐3‐one and 5β‐cholestane‐3α,7α,12α,25‐tetrol‐3‐*O*‐β‐d‐glucuronide were previously demonstrated as useful for screening newborn dried blood‐spots for CTX^9^, ^10^, ^11^ and may be part of a future screening algorithm. Although these compounds were elevated in the CTX cases described here, additional studies may be necessary to determine whether this subset of patients is readily detected by proposed newborn screening methods.

In summary, the two patients reported here are the first known cases of CTX patients with normal cholestanol levels that may represent a milder biochemical form of CTX. One sibling appeared to have no neurological involvement at age 60, with mild symptoms of disease, the other sibling had severe clinical symptoms with onset of spastic paraparesis beginning in the third decade of life. In a disorder such as CTX with a heterogeneous clinical presentation and long diagnostic lag time, the possibility of cases that are not detected by standard biochemical methods offers another diagnostic hurdle that must be considered. Fortunately, biochemical panels including bile acid precursors and molecular analysis of *CYP27A1* are available and offer alternative testing methods to direct measurement of cholestanol. In light of the patients presented here, biochemical panels including bile acid precursors should be ordered in addition to plasma cholestanol for cases of suspected CTX.

## CONFLICT OF INTEREST

A. D. has received Honoraria from Leadiant Biosciences and Retrophin, as well as grant funding from Retrophin. The OHSU Foundation and Chemical Physiology and Biology Department have received gifts from Retrophin. These gifts, which have not been made specifically in connection with this research, have been reviewed by the OHSU integrity office. All other authors declare no conflict of interest.

## AUTHOR CONTRIBUTIONS

**Adam J. Guenzel**: Designed the study and wrote the initial draft of the manuscript. **Andrea DeBarber**: Performed additional testing on the patient samples and participated in manuscript preparation and review. **Kimiyo Raymond**: Was responsible for development and analysis of the biochemical assays used to identify these patients. **Radhika Dhamija**: Was the geneticist that facilitated testing and diagnosed both patients with CTX and also obtained informed consent.

## COMPLIANCE WITH ETHICS AND GUIDELINES

All procedures followed were in accordance with the ethical standards of the responsible committee on human experimentation (institutional and national) and with the Helsinki Declaration of 1975, as revised in 2000 (5). Informed consent was obtained from all patients for being included in the study.

## PATIENT CONSENT

This article does not contain any studies with human or animal subjects performed by the any of the authors.
